# New Appliances and Things Medical

**Published:** 1910-08-20

**Authors:** 


### NEW APPLIANCES AND THINGS MEDICAL.

[We shall be glad to receive at our Office, 28 & 29 Southampton Street, Strand, London, W.C., from the manufacturers, specimens of all new preparations and appliances.]

### THE WINCARNIS STETHOSCOPE.

(Coleman and Co., Ltd., Wincarnis Works, Norwich.)

In general the profession is quite properly somewhat shy of the various new stethoscopes of unfamiliar shapes and high-sounding names which are now and then put upon the market. Even the old wooden stethoscope still holds its own against the more modern binaural pattern, and some eminent consulting physicians can actually discern more with the old instrument if perfect quiet is maintained in the vicinity. Three minutes, however, will be sufficient to convince anyone that, in the novel machine which Messrs. Coleman have just introduced, an instrument of perfectly amazing resonating and sound-collecting properties is offered to the profession. Without exaggeration it may be affirmed that the cardiac and pulmonary sounds can be perceived *through the clothing* with as much (or even more) distinctness as on the bare chest with the usual patterns. There is, moreover, a crisp, almost musical, *timbre* about the conducted sounds which renders very simple and certain the detection of any murmur or other pathological sign. This is advantage number one, and a very important one; the next point to which the manufacturers draw attention is its lightness and portability. The whole apparatus goes into a small celluloid box as big as an eighteenth-century watch, and its weight is, perhaps, a quarter of that of the ordinary binaural stethoscope. Lastly, the chest-piece is capable of being detached and thrown away, to be replaced by another of simple and cheap construction, thus rendering the instrument available for infective cases with the minimum of trouble over disinfection.

In construction the stethoscope is simplicity itself. A thin wooden disc forms the chest-piece; it fits into a metal rim which secures it, and is perforated by a central aperture, which is made of varying size to suit various cases. Behind the aperture in the disc is a plate of mica fixed by a small brass screw so as to lie parallel to the chest-piece, and parallel also to the (wooden) back of the instrument, to which the ear-tubes are attached. By loosening or tightening the nut of this screw the tension of the mica plate can be decreased or increased, and its sensitiveness to sounds of low or high pitch varied. With the nut fairly tight the aortic second sound can be heard, through one's ordinary clothes, as a clear, bell-like note, which is most reassuring as to the state of one's own aortic valves. Messrs. Coleman have introduced a stethoscope which is really an advance on anything yet invented, and medical men may be advised to test for themselves one of these new stethoscopes at the first opportunity.

### AN ANTISEPTIC EAR-PIERCER.

(Calipe, Dettmer & Co., 21 Poland Street, W.)

The little surgical operation which fashion decrees for those ladies who desire to wear old-fashioned ear-rings is essentially a minor operation, and yet it is unfortunately true that, like other minor operations, it has sometimes cost the patient who submitted to it very serious inconvenience and trouble. Some fatal results have been chronicled; in one case owing to severe erysipelas, and in another due to spreading cellulitis. Anyone who undertakes to pierce a lady's ear for "ringing" should therefore take the ordinary antiseptic or aseptic precautions which are an imperative preliminary of every operation. We have recently had submitted to us an ingenious antiseptic ear-piercer manufactured by the above firm, which is in every way worthy the attention of the general practitioner, whose advice is sometimes asked with regard to this trifling ritual of fashion. It consists of an automatic spring needle, released by a trigger and working between two sterilised corks, which firmly maintain the lobule of the ear in position. The whole apparatus may be sterilised by dry heat or by boiling, and the tubes which are supplied to keep the hole patent are sterilised in carbolic oil. The piercer is supplied in a neat little case, together with tubes, spare needles, a tube of 25 per cent. phenol (Bobœuf), and another containing accessory sterilised corks, new corks being, of course, used for each patient. The contrivance is simple, strong, and effective, and may be obtained on application to the firm at the above address.

### THERMOGENE.

(The Thermogene Company, Ltd., Haywards Heath, Sussex.)

Thermogene is already well known to most practitioners, but it deserves to be much more widely known, for its virtues are manifold. We would suggest to the makers that they should issue a brief booklet to accompany their wares and draw attention to the various ways in which this absorbent wool wadding can be utilised. Many practitioners will doubtless be ready to state one or two cases for their benefit. We have lately found it of decided use in combination with passive congestion in cases of rheumatoid arthritis, and we have heard from a colleague of its value in cases of alcoholic cirrhosis. In the latter the wool is moistened with a dilute solution of hydrochloric acid, and is probably more efficacious than the old acid poultice which at one time enjoyed a decided popularity. Personally we have found thermogene of use in quinsy and tonsilitis; a roll of the wadding, slightly warmed, sprinkled with a few drops of some scent, and lightly tied round the throat with a towel, gives great relief from pain in these conditions and is a valuable adjuvant to treatment. The advantages of thermogene are that it is clean, safe, easy to apply, and cheap, and that it lasts a comparatively long time. Every practitioner should keep it in stock, and it should be listed as one of the indispensable necessities in every first-aid outfit. The special rates quoted for quantities may be obtained on application to the makers.

The sub-committee of the Johannesburg Hospital Board has reported on the recent confusion at the hospital mortuary—as a result of which the remains of one lady were interred as those of another. The report finds that there was carelessness, and severely reprimands the mortuary attendant.

THE HOSPITAL. August 20, 1910.

